# Prevalence and in-hospital outcomes of diabetes among patients with acute coronary syndrome in China: findings from the Improving Care for Cardiovascular Disease in China-Acute Coronary Syndrome Project

**DOI:** 10.1186/s12933-018-0793-x

**Published:** 2018-11-27

**Authors:** Mengge Zhou, Jing Liu, Yongchen Hao, Jun Liu, Yong Huo, Sidney C. Smith, Junbo Ge, Changsheng Ma, Yaling Han, Gregg C. Fonarow, Kathryn A. Taubert, Louise Morgan, Na Yang, Yueyan Xing, Dong Zhao, the CCC-ACS Investigators

**Affiliations:** 1 Department of Epidemiology, Beijing Anzhen Hospital, The Key Laboratory of Remodeling-Related Cardiovascular Diseases, Ministry of Education, Beijing Municipal Key Laboratory of Clinical Epidemiology, Beijing Institute of Heart, Lung and Blood Vessel Diseases, Capital Medical University, No. 2 Anzhen Road, Chaoyang District, Beijing, 100029 China; 2 Department of Cardiology, Peking University First Hospital, Beijing, China; 3 Division of Cardiology, University of North Carolina, Chapel Hill, NC USA; 4 Department of Cardiology, Shanghai Institute of Cardiovascular Diseases, Zhongshan Hospital, Fudan University, Shanghai, China; 5 Department of Cardiology, Beijing Anzhen Hospital, Capital Medical University, Beijing, China; 6 Cardiovascular Research Institute and Department of Cardiology, General Hospital of Shenyang Military Region, Shenyang, Liaoning China; 7 Division of Cardiology, Geffen School of Medicine at University of California, Los Angeles, CA USA; 8 Department of International Science, American Heart Association, Basel, Switzerland; 9 International Quality Improvement Department, American Heart Association, Dallas, TX USA

**Keywords:** Acute coronary syndrome, Diabetes, Prevalence, Epidemiology, Death, MACCE, In-hospital outcome, CCC-ACS

## Abstract

**Background:**

Guidelines have classified patients with acute coronary syndrome (ACS) and diabetes as a special population, with specific sections presented for the management of these patients considering their extremely high risk. However, in China up-to-date information is lacking regarding the burden of diabetes in patients with ACS and the potential impact of diabetes status on the in-hospital outcomes of these patients. This study aims to provide updated estimation for the burden of diabetes in patients with ACS in China and to evaluate whether diabetes is still associated with excess risks of early mortality and major adverse cardiovascular and cerebrovascular events (MACCE) for ACS patients.

**Methods:**

The Improving Care for Cardiovascular Disease in China-ACS Project was a collaborative study of the American Heart Association and the Chinese Society of Cardiology. A total of 63,450 inpatients with a definitive diagnosis of ACS were included. Prevalence of diabetes was evaluated in the overall study population and subgroups. Multivariate logistic regression was performed to examine the association between diabetes and in-hospital outcomes, and a propensity-score-matched analysis was further conducted.

**Results:**

Among these ACS patients, 23,880 (37.6%) had diabetes/possible diabetes. Both STEMI and NSTE-ACS patients had a high prevalence of diabetes/possible diabetes (36.8% versus 39.0%). The prevalence of diabetes/possible diabetes was higher in women (45.0% versus 35.2%, *p *< 0.001). Even in patients younger than 45 years, 26.9% had diabetes/possible diabetes. While receiving comparable treatments for ACS, diabetes/possible diabetes was associated with a twofold higher risk of all-cause death (adjusted odds ratio 2.04 [95% confidence interval 1.78–2.33]) and a 1.5-fold higher risk of MACCE (adjusted odds ratio 1.54 [95% confidence interval 1.39–1.72]).

**Conclusions:**

Diabetes was highly prevalent in patients with ACS in China. Considerable excess risks for early mortality and major adverse cardiovascular events were found in these patients.

*Trial registration* NCT02306616. Registered December 3, 2014

**Electronic supplementary material:**

The online version of this article (10.1186/s12933-018-0793-x) contains supplementary material, which is available to authorized users.

## Background

Patients with both clinical cardiovascular disease (CVD) and diabetes were classified as extreme-risk groups in recently published guidelines issued by the American Association of Clinical Endocrinologists and the American College of Endocrinology [1]. The latest guidelines for ST-segment elevation myocardial infarction (STEMI) and non-ST-segment elevation acute coronary syndrome (NSTE-ACS) also classified patients with acute coronary syndrome (ACS) and diabetes as a special population and presented specific sections for the management of these patients in consideration of their extremely high risk [2–5]. However, limited studies have been conducted to evaluate the burden of diabetes on ACS patients in China in recent years. The latest studies to focus on the prevalence of diabetes were conducted more than 10 years ago [6, 7]. With the rapid increase in the prevalence of diabetes among the general population in China, the burden of diabetes among Chinese ACS patients needs to be re-evaluated. In addition, despite the advancements in the clinical management and the wide application of percutaneous coronary intervention (PCI) in the past decade, whether the excess risk caused by diabetes is reduced among ACS has remained unclear. Therefore, an up-to-date evaluation regarding the prevalence of diabetes in ACS patients in China and the potential impact of diabetes status on the outcomes of these patients during hospitalization is needed.

In this study, we aim to provide an updated estimation of the burden of diabetes in patients with ACS and to evaluate whether diabetes is still associated with excess risks for in-hospital all-cause death or major adverse cardiovascular and cerebrovascular events (MACCE) to these patients in China, based on the Improving Care for Cardiovascular Disease in China-ACS Project (CCC-ACS Project), a large nationwide registry and quality improvement study.

## Research design and methods

### Study design and population

The CCC-ACS project, a nationwide registry and quality improvement study with an ongoing database focusing on quality of ACS care, was launched in 2014 as a collaborative initiative of the American Heart Association and the Chinese Society of Cardiology. In Phases I and II of the project, only the tertiary hospitals were included, 150 centers representing the diversity of care for ACS in tertiary hospitals across China. Since July 2017, Phase III of the project has extended into secondary hospitals. The data for this study are based on Phases I and II of the project. Details of the design and methodology of the CCC project have been published [8]. A standard web-based data collection platform (Oracle Clinical Remote Data Capture, Oracle) was used in this study. Trained data abstractors in the participating hospitals reported the required data, which they abstracted from the patients’ original medical records. Eligible patients were consecutively reported to the CCC-ACS database for each month before the middle of the following month. Third-party clinical research associates performed quality audits to ensure that cases were reported consecutively rather than selectively. In addition, about 5% of reported cases were randomly selected, and the reported information was compared with the original medical records as a quality assessment and a method to promote accuracy and completeness of the reported data. According to the quality audit reports, the data in this study were appropriately reported with an accuracy rate greater than 95%.

Based on principal discharge diagnosis, 63,641 inpatients with ACS were registered between November 2014 and June 2017 from 150 hospitals. Of these, 63,450 inpatients were included in this study after excluding 191 (0.3%) patients with incomplete demographic information. The flow chart for study population recruitment can be found in Additional file 1: Figure S1.

### Definition of diabetes

Diabetes was defined according to one of the following criteria: (1) a self-reported diabetes which was previously diagnosed by physicians or use of glucose-lowering drugs before hospitalization; (2) diabetes listed in the medical records as the secondary discharge diagnosis; (3) glycated hemoglobin A1c (HbA1c) concentration ≥ 6.5%.

Possible diabetes was defined in ACS patients with level of fasting blood glucose (FBG) ≥ 7.0 mmol/L but without measurement of HbA1c, as we could not distinguish between undiagnosed diabetes and stress hyperglycemia in this group of patients by the results of FBG alone.

### Definition of in-hospital outcomes

The outcomes of this study included all-cause deaths and MACCEs that occurred during hospitalization. MACCEs were defined as a combination of cardiac death, recurrent myocardial infarction, stent thrombosis, and stroke. All of these outcomes were diagnosed by doctors during patients’ hospitalization and recorded in medical records.

### Definition of other variables

Hypertension was defined as having a history of hypertension, receiving antihypertensive therapy, or systolic blood pressure (SBP) ≥ 140 mmHg or diastolic blood pressure (DBP) ≥ 90 mmHg at admission. Elevated low-density lipoprotein cholesterol (LDL-C) was defined as serum LDL-C ≥ 1.8 mmol/L (70 mg/dL). Low high-density lipoprotein cholesterol (HDL-C) was defined as serum HDL-C < 1.0 mmol/L (40 mg/dL). Elevated triglyceride (TG) was defined as serum TG ≥ 2.3 mmol/L (200 mg/dL). Current smoking was defined as smoking in the preceding 1 year according to the medical records of the patients. Estimated glomerular filtration rate (eGFR) was calculated by the equation developed by the Chronic Kidney Disease Epidemiology Collaboration [9]. A history of coronary heart disease (CHD) was specified if the patients had a clinical history of myocardial infarction or underwent PCI or coronary artery bypass grafting (CABG) before the current hospitalization. Other clinical history of diseases, including cerebrovascular disease, heart failure, peripheral artery disease (PAD), atrial fibrillation, and renal failure was defined according to the notes on original medical records. Heart failure, cardiac arrest, and cardiac shock occurring within 24 h of the current admission were defined as a severe clinical condition. The definition of fivefold elevated myocardial injury markers was elevation of cardiac injury marker beyond fivefold the upper reference limit [5]. In addition, we evaluated the risk of in-hospital death using the Global Registry of Acute Coronary Events (GRACE) score whereby patients with a score greater than 140 were classified as high risk [10]. Subtypes of ACS were defined based on the principal discharge diagnosis of medical records. Patients with a diagnosis of non-STEMI and unstable angina were classified as NSTE-ACS. Cardiologists diagnosed patients based on guidelines of STEMI and NSTE-ACS issued by the Chinese Society of Cardiology [11, 12]. The diagnostic criteria included symptoms of chest pain, results of ECG, and biomarkers of myocardial injury.

### Statistical analysis

As most of the patients with possible diabetes could be undiagnosed or were at high risk of developing diabetes, and needed the same care as diabetic patients during hospitalization [2–5, 13], for the purposes of this study we combined diabetes and possible diabetes for analysis. Prevalence of diabetes/possible diabetes and its 95% confidence intervals (CIs) were estimated in the overall study population and in subgroups by sex, age groups, and CHD history. The characteristics, in-hospital treatments, and in-hospital outcomes of these patients were described and compared according to diabetic status in ACS patients. Continuous variables with normal distribution were shown as mean (standard deviation [SD]) and differences between groups were compared using *t*-tests; continuous variables with skewed distribution were shown as median (interquartile range [IQR]) and compared using the Mann–Whitney U test; and categorical variables were presented as the number (percentage) and compared using chi-square test. Logistic multivariable regression analysis was carried out to examine the association between diabetes/possible diabetes and in-hospital outcomes. Univariate analysis was performed first, followed by multivariate-adjusted analysis. The candidate adjusted factors are confounding factors that either have been included in the risk assessment or have been reported more than once with an effect on death or MACCE, including baseline characteristics, risk factors, medical history, clinical conditions at admission, and treatment during hospitalization, i.e., age (continuous), sex (male/female), current smoking (yes/no), SBP levels (continuous), heart rate (continuous), cardiac arrest at admission (yes/no), Killip class at admission (class I/II–III/IV), history of CHD (yes/no), cerebrovascular disease (yes/no), PAD (yes/no), heart failure (yes/no), renal failure (yes/no), eGFR (continuous), administration of dual antiplatelet therapy (yes/no), anticoagulant therapy (yes/no), statins (yes/no), β-blockers (yes/no), and angiotensin-converting enzyme inhibitors (ACEIs)/angiotensin-receptor blockers (ARBs) (yes/no) during hospitalization, PCI treatment (yes/no), fivefold elevated myocardial injury markers (yes/no), type of ACS (STEMI/NSTE-ACS), and whether patients were transferred from another hospital before the current hospitalization (yes/no). After forward stepwise selection with entry and exit criteria both set at the *p *= 0.15 level, the variables listed in the legend of Table 4 were eventually included in the multivariable adjusted logistic model of all-cause death and MACCE, respectively. Given the differences in pathologies, management, and prognosis of STEMI and NSTE-ACS, we performed the above analyses in these two subtypes of ACS patients.

Since some ACS patients with FBG ≥ 7.0 mmol/L but with HbA1c < 6.5% were classified as patients without diabetes, who could mostly be diagnosed with stress hyperglycemia and associated with increased risk of death and MACCE, we conducted a sensitivity analysis by excluding these patients and recalculated the risk of diabetes/possible diabetes.

Subgroup analysis, including age, sex, Killip class, eGFR, GRACE score, PCI treatment, types of ACS, and whether the patient was transferred before the current hospitalization, was performed by using important characteristics in a multivariable adjusted logistic regression model. Odds ratios (ORs) between subgroups were compared using a *Z*-test [14].

In addition, we conducted a propensity-score-matched analysis to further confirm the association between diabetes/possible diabetes and in-hospital outcomes. First, a propensity score of having diabetes/possible diabetes was calculated by a logistic regression model with the variables age, sex, SBP levels, heart rate, LDL-C, HDL-C, TG, eGFR, Killip class at admission, history of myocardial infarction, PCI, CABG, cerebrovascular disease, heart failure, PAD, atrial fibrillation, renal failure, and type of ACS. Patients with and without diabetes/possible diabetes were then matched at a 1:1 ratio by propensity score using nearest-neighbor matching without replacement, with a caliper of 0.02. The absolute standardized differences of variables included for the calculation of propensity score were compared before and after propensity-score matching. Standardized differences < 10.0% for these included variables indicated a relatively small imbalance. The baseline characteristics and in-hospital management between the two propensity-score-matched subsets were re-compared. As some characteristics did not exactly match between the two groups even after the propensity-score matching, multivariable logistic regression was further performed to compare the risk by adjusting factors eventually included in the whole study population by stepwise selection.

Statistical analyses were performed using SAS 9.4 (SAS Institute, Cary, NC, USA) and Stata 14.0 (Stata, College Station, TX, USA). Two-tailed *p* values of less than 0.05 were considered statistically significant.

## Results

### Prevalence of diabetes in ACS patients

The average age of the 63,450 ACS patients, 25.1% of whom were female, was 62.9 (± 12.4) years. Among these patients, a total of 23,880 (37.6%) had diabetes/possible diabetes (Table 1), including 29.7% diabetes and 7.9% possible diabetes. Both STEMI and NSTE-ACS patients had a high prevalence of diabetes/possible diabetes, but prevalence was slightly higher in patients with NSTE-ACS than in those with STEMI (39.0% versus 36.8%). Women had a higher proportion of diabetes/possible diabetes than men (45.0% versus 35.2%). The prevalence of diabetes/possible diabetes increased significantly with age. However, even in patients younger than 45 years, 26.9% of them had diabetes/possible diabetes. Patients with a history of CHD had a higher prevalence of diabetes/possible diabetes than those without CHD history (45.9% versus 36.6%).

**Table 1:** Prevalence of diabetes/possible diabetes in patients with ACS

	Total ACS (*N *= 63,450)	STEMI (*N *= 39,793)	NSTE-ACS (*N *= 23,657)
Total, n (% [95% CI])	23,880 (37.6 [37.3–38.0])	14,650 (36.8 [36.3–37.3])	9230 (39.0 [38.4–39.6])
Sex, n (% [95% CI])			
Male	16,721 (35.2 [34.8–35.6])	10,746 (34.5 [34.0–35.0])	5975 (36.5 [35.7–37.2])
Female	7159 (45.0 [44.2–45.7])	3904 (45.1 [44.1–46.2])	3255 (44.7 [43.6–45.9])
Age, n (% [95% CI]) (years)			
< 45	1281 (26.9 [25.6–28.1])	1003 (27.5 [26.1–29.0])	278 (24.7 [22.2–27.2])
45–64	10,726 (36.2 [35.6–36.7])	7041 (36.1 [35.4–36.8])	3685 (36.2 [35.3–37.2])
≥ 65	11,873 (40.9 [40.4-41.5])	6606 (39.7 [38.9-40.4])	5267 (42.6 [41.8–43.5])
CHD history, n (% [95% CI])			
Yes	3197 (45.9 [44.7–47.1])	1186 (44.3 [42.4–46.2])	1186 (44.3 [42.4–46.2])
No	20,683 (36.6 [36.2–37.0])	13,464 (36.3 [35.8–36.8])	7219 (37.3 [36.6–38.0])

*CHD* coronary heart disease, *ACS* acute coronary syndrome, *STEMI* ST-segment elevation myocardial infarction, *NSTE*-*ACS* non-ST-segment elevation acute coronary syndrome

### Characteristics of ACS patients with diabetes

Compared with ACS patients without diabetes, patients with diabetes/possible diabetes had a higher frequency of previous diseases and major cardiovascular risk factors (Table 2). Of these ACS patients with diabetes/possible diabetes, 21.5% had previously diagnosed CVD and 51.2% had three or more other cardiovascular risk factors, including hypertension, different types of dyslipidemia, and smoking.

**Table 2:** Characteristics of ACS patients with diabetes/possible diabetes

	All ACS			STEMI			NSTE-ACS		
	Diabetes/possible diabetes (N = 23,880)	No diabetes (N = 39,570)	*p* value	Diabetes/possible diabetes (N = 14,650)	No diabetes (N = 25,143)	*p* value	Diabetes/possible diabetes (N = 9230)	No diabetes (N = 14,427)	*p* value
Age, mean (SD), years	64.2 (11.9)	62.1 (12.7)	< 0.001	62.9 (12.0)	61.0 (12.9)	< 0.001	66.3 (11.3)	64.1 (12.2)	< 0.001
Women, n (%)	7159 (30.0)	8764 (22.2)	< 0.001	3904 (26.7)	4744 (18.9)	< 0.001	3255 (35.3)	4020 (27.9)	< 0.001
Vital signs									
SBP levels, mean (SD)	131.7 (24.3)	129.1 (22.9)	< 0.001	128.4 (24.2)	126.3 (22.9)	< 0.001	136.9 (23.5)	133.9 (22.1)	< 0.001
DBP levels, mean (SD)	77.9 (14.5)	78.0 (14.3)	0.543	77.8 (15.0)	77.5 (14.6)	0.629	78.4 (13.5)	78.8 (13.6)	0.040
Heart rate, mean (SD)	79.6 (17.2)	76.1 (15.5)	< 0.001	80.0 (17.5)	76.8 (15.8)	< 0.001	79.1 (16.6)	74.8 (14.8)	< 0.001
Risk factors									
Hypertension, n (%)	17,180 (71.9)	24,176 (61.1)	< 0.001	9961 (68.0)	14,350 (57.1)	< 0.001	7219 (78.2)	9826 (68.1)	< 0.001
Elevated LDL-C^a^, n (%)	18,024 (83.7)	30,517 (85.1)	0.010	11,375 (86.5)	19,760 (86.9)	0.257	6649 (79.3)	10,757 (82.0)	< 0.001
Low HDL-C^b^, n (%)	10,634 (49.1)	15,238 (42.4)	< 0.001	6293 (47.6)	9768 (42.8)	< 0.001	4341 (51.5)	5470 (41.6)	< 0.001
Elevated TG^c^, n (%)	5194 (23.9)	5911 (16.4)	< 0.001	3094 (23.3)	3659 (16.0)	< 0.001	2100 (24.9)	2252 (17.1)	< 0.001
Current smoker, n (%)									
Men	8499 (50.8)	17,421 (56.6)	< 0.001	5848 (54.4)	12,081 (59.2)	< 0.001	2651 (44.4)	5340 (51.3)	< 0.001
Women	572 (8.0)	851 (9.7)	< 0.001	371 (9.5)	547 (11.5)	0.002	201 (6.2)	304 (7.6)	0.021
History of diseases									
CHD, n (%)	3197 (13.4)	3766 (9.5)	< 0.001	1186 (8.1)	1491 (5.9)	< 0.001	2011 (21.8)	2275 (15.8)	< 0.001
Cerebrovascular disease, n (%)	2911 (12.2)	3200 (8.1)	< 0.001	1587 (10.8)	1850 (7.4)	< 0.001	1324 (14.3)	1350 (9.4)	< 0.001
Heart failure, n (%)	706 (3.0)	553 (1.4)	< 0.001	199 (1.4)	178 (0.7)	< 0.001	507 (5.5)	375 (2.6)	< 0.001
Atrial fibrillation, n (%)	674 (2.8)	855 (2.2)	< 0.001	253 (1.7)	328 (1.3)	0.001	421 (4.6)	527 (3.7)	0.001
PAD, n (%)	293 (1.2)	325 (0.8)	< 0.001	102 (0.7)	145 (0.6)	0.143	191 (2.1)	180 (1.3)	<0.001
Renal failure, n (%)	645 (2.7)	423 (1.1)	< 0.001	237 (1.6)	188 (0.8)	< 0.001	408 (4.4)	235 (1.6)	< 0.001
Severe clinical conditions									
Heart failure^d^, n (%)	2805 (11.9)	2813 (7.2)	< 0.001	1683 (11.6)	1816 (7.3)	< 0.001	1122 (12.3)	997 (7.0)	< 0.001
Cardiac shock^e^, n (%)	874 (3.7)	1049 (2.7)	< 0.001	728 (5.0)	892 (3.6)	< 0.001	146 (1.6)	157 (1.1)	0.001
Cardiac arrest^f^, n (%)	522 (2.2)	693 (1.8)	< 0.001	427 (2.9)	605 (2.4)	0.005	95 (1.0)	88 (0.6)	< 0.001
Killip class^g^, n (%)			< 0.001			< 0.001			< 0.001
II–III	6700 (28.1)	9406 (23.8)		3924 (26.9)	5870 (23.4)		2776 (30.2)	3536 (24.6)	
IV	1569 (6.6)	1725 (4.4)		1147 (7.9)	1325 (5.3)		422 (4.6)	400 (2.8)	
Fivefold elevated myocardial injury marker^h^	16,800 (72.7)	26,759 (70.5)	< 0.001	11,779 (82.6)	19,638 (81.0)	< 0.001	5021 (56.8)	7121 (51.9)	< 0.001
GRACE score ≥ 140^i^, n (%)	8632 (39.2)	11,196 (31.2)	< 0.001	9074 (65.7)	16,819 (72)	< 0.001	3887 (47.3)	4644 (37.0)	< 0.001
Type of ACS, n (%)			< 0.001						
STEMI	14,650 (61.4)	25,143 (63.5)		–	–	–	–	–	–
NSTE-ACS	9230 (38.7)	14,427 (36.5)		–	–	–	–	–	–
Patients with referral, n (%)	10,148 (42.5)	18,396 (46.5)	< 0.001	7198 (49.2)	13,347 (53.1)	< 0.001	2950 (32.0)	5049 (35.0)	< 0.001

*ACS* acute coronary syndrome, *LDL*-*C* low-density lipoprotein cholesterol, *HDL*-*C* high-density lipoprotein cholesterol, *TG* triglyceride, *CHD* coronary heart disease, *PAD* peripheral artery disease, *GRACE* Global Registry of Acute Coronary Events, *ACS* acute coronary syndrome, *STEMI* ST-segment elevation myocardial infarction, *NSTE*-*ACS* non-ST-segment elevation acute coronary syndrome

^a^ Elevated LDL-C, data of LDL-C were not available for 6060 patients

^b^ Decreased HDL-C, data of HDL-C were not available for 5860 patients

^c^ Elevated TG, data of TG were not available for 5614 patients

^d^ Heart failure, data of heart failure were not available for 766 patients

^e^ Cardiac shock, data of cardiac shock were not available for 743 patients

^f^ Cardiac arrest, data of cardiac arrest were not available for 807 patients

^g^ Killip class, data of first Killip class were not available for 181 patients

^h^ Fivefold elevated myocardial injury markers, data of myocardial injury markers were not available for 2390 patients

^i^ GRACE score, data of cardiac arrest were not available for 5504 patients

ACS patients with diabetes/possible diabetes also had more severe clinical conditions than those without diabetes at admission, with a higher frequency of heart failure (11.9% versus 7.2%), cardiac shock (3.7% versus 2.7%), and cardiac arrest (2.2% versus 1.8%). In addition, the proportion of high-risk patients based on GRACE scores was also significantly higher in patients with diabetes/possible diabetes in comparison with non-diabetic patients (39.2% versus 31.2%). Similar results were observed in subtypes of ACS patients with and without diabetes/possible diabetes.

### In-hospital management of ACS patients with diabetes

We compared the treatments for ACS between patients with and without diabetes/possible diabetes with regard to STEMI and NSTE-ACS (Table 3). Most of the received treatments, including PCI, antiplatelet therapy, anticoagulant therapy, statins, and β-blockers, were comparable between patients with and without diabetes/possible diabetes. We did not observe a higher rate of CABG in ACS patients with diabetes in this study.

**Table 3:** In-hospital management of ACS patients with diabetes/possible diabetes

	STEMI			NSTE-ACS		
	Diabetes/possible diabetes (N = 14,650)	No diabetes (N = 25,143)	*p* value	Diabetes/possible diabetes (N = 9230)	No diabetes (N = 14,427)	*p* value
DAPT, % (n/N)	95.4 (13,911/14,577)	95.5 (23,915/25,037)	0.686	89.4 (8130/9098)	90.0 (12,827/14,250)	0.108
Aspirin, % (n/N)	96.3 (14,032/14,579)	96.5 (24,163/25,038)	0.184	93.1 (8469/9100)	93.6 (13,343/14,253)	0.099
P2Y_12_ inhibitors, % (n/N)	96.9 (14,194/14,643)	96.7 (24,295/25,135)	0.134	92.7 (8534/9211)	92.9 (13,394/14,411)	0.395
GPIIb/IIIa, % (n/N)	39.8 (5816/14,625)	38.4 (9651/25,112)	0.008	17.5 (1612/9196)	17.3 (2494/14,406)	0.668
Anticoagulant, % (n/N)	79.4 (11,632/14,643)	79.2 (19,912/25,135)	0.606	68.1 (6273/9211)	67.1 (9668/14,411)	0.104
UFH	5.3 (768/14,623)	4.5 (1121/25,097)	< 0.001	2.0 (185/9171)	1.9 (271/14,379)	0.472
LMWH	73.0 (10,676/14,623)	73.7 (18,490/25,097)	0.147	62.8 (5760/9171)	62.4 (8969/14,379)	0.505
Fondaparinux sodium	1.3 (186/14,623)	1.0 (262/25,097)	0.038	2.2 (201/9171)	1.6 (232/14,379)	0.001
Other anticoagulants	1.9 (280/14,623)	2.0 (512/25,097)	0.389	2.0 (185/9171)	2.0 (280/14,379)	0.707
Statins, % (n/N)	94.1(37,382/39,735)	94.0 (13,748/14,628)	0.544	92.9 (8562/9212)	93.4 (13,459/14,408)	0.161
Beta-blockers, % (n/N)	64.3 (2917/4536)	62.5 (4998/7997)	0.044	67.6 (2046/3028)	66.4 (3431/5164)	0.295
ACEI/ARB, % (n/N)	49.8 (6687/13,427)	48.4 (11,151/23,030)	0.011	54.0 (4643/8606)	48.8 (6535/13,396)	< 0.001
PCI, % (n/N)	77.1 (11,289/14,650)	77.5 (19,484/25,143)	< 0.001	58.2 (5367/9230)	61.1 (8810/14,427)	0.006
Time of PCI^a^, % (n/N) (h)			< 0.001			0.006
< 2	55.2 (8574/15,526)	57.4 (5220/9100)		10.0 (734/7341)	8.9 (388/4383)	
2–11.9	10.2 (1577/15,526)	11.5 (1046/9100)		10.0 (731/7341)	10.8 (474/4383)	
12–23.9	3.2 (493/15,526)	3.4 (313/9100)		7.8 (572/7341)	7.2 (314/4383)	
24–71.9	9.8 (1524/15,526)	8.9 (809/9100)		31.2 (2293/7341)	29.5 (1294/4383)	
≥ 72	21.6 (3358/15,526)	18.8 (1712/9100)		41.0 (3011/7341)	43.7 (1913/4383)	
Type of stents^b^, % (n/N)			0.010			0.088
Drug eluting stent	98.1 (9827/10,017)	98.1 (16,825/17,145)		96.9 (4317/4456)	96.7 (6997/7235)	
Bare metal stent	1.0 (101/10,017)	1.2 (213/17,145)		1.8 (82/4456)	1.6 (113/7235)	
Other	0.9 (89/10,017)	0.6 (107/17,145)		1.3 (57/4456)	1.7 (125/7235)	
CABG, % (n/N)	0.5 (46/10,121)	0.5 (94/17,328)	0.324	0.7 (33/4670)	0.7 (51/7583)	0.824
Length of stay, median (IQR), day	10.0 (7.0–13.0)	10.0 (7.0–13.0)	< 0.001	10.0 (7.0–13.0)	9.0 (7.0–12.0)	< 0.001

*STEMI* ST-segment elevation myocardial infarction, *NSTE*-*ACS* non-ST-segment elevation acute coronary syndrome, *DAPT* dual antiplatelet therapy, *UFH* unfractionated heparin, *LMWH* low molecular weight heparin, *PCI* percutaneous coronary intervention, *CABG* coronary artery bypass grafting

The usage rate of drugs was calculated in patients without drug contraindications

^a^ Time of PCI, time from admission to PCI, and detailed data of time of PCI were not available for 8600 patients with PCI

^b^ Type of stents and type of PCI were not available for 849 patients with stent implantation

### In-hospital outcomes of ACS patients with diabetes

**Fig. 1:**
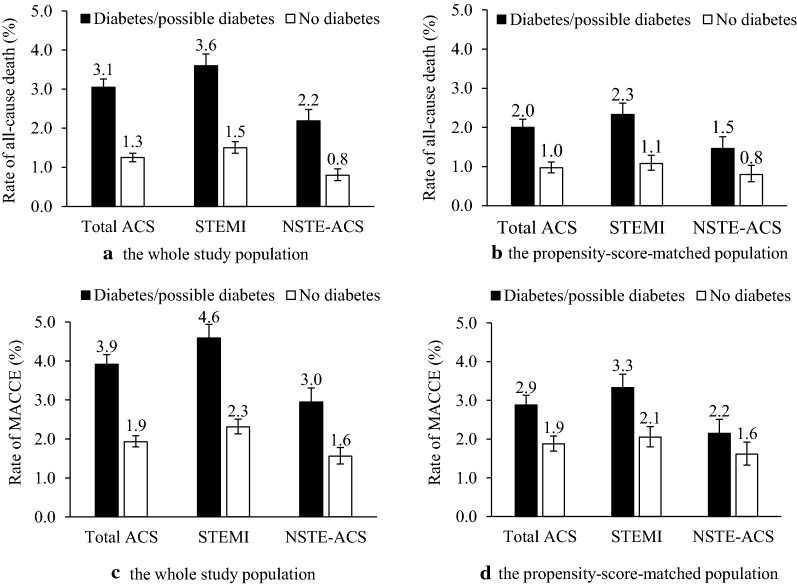
In-hospital outcomes of ACS patients with and without diabetes. **a** In-hospital all-cause death of the whole study population. **b** In-hospital all-cause death of the propensity-score-matched population. **c** In-hospital MACCE of the whole study population. **d** In-hospital MACCE of the propensity-score-matched population. *MACCE* major adverse cardiovascular and cerebrovascular events

The in-hospital outcomes were compared between ACS patients with and without diabetes/possible diabetes (Fig. 1 and Additional file 1: Table S1). Higher rates of all-cause death and MACCE were observed in all ACS patients with diabetes/possible diabetes as well as in the subtypes of ACS. In univariate logistic regression analysis, a significantly higher risk of all-cause death and MACCE was observed in patients with diabetes/possible diabetes (Table 4). The independent association was further evaluated using multivariable analyses (Table 4 and Additional file 1: Tables S2 and S3). After multivariable adjustment, diabetes/possible diabetes was associated with a twofold increased risk of all-cause death (OR, 2.04 [95% CI 1.78–2.33]) and a 1.5-fold increased risk of MACCE (OR, 1.54 [95% CI 1.39–1.72]).

**Table 4:** Association between diabetes/possible diabetes and in-hospital outcomes

	All ACS				STEMI				NSTE-ACS			
	Unadjusted OR (95% CI)	*p* value	Adjusted OR (95% CI)	*p* value	Unadjusted OR (95% CI)	*p* value	Adjusted OR (95% CI)	*p* value	Unadjusted OR (95% CI)	*p* value	Adjusted OR (95% CI)	*p* value
The whole study population												
All-cause death^a^	2.49 (2.22–2.80)	< 0.001	2.04 (1.78–2.33)	< 0.001	2.45 (2.14–2.80)	< 0.001	2.07 (1.76–2.43)	< 0.001	2.76 (2.19–3.47)	< 0.001	1.93 (1.48–2.51)	< 0.001
MACCE^b^	1.98 (1.80–2.18)	< 0.001	1.54 (1.39–1.72)	< 0.001	2.03 (1.81–2.27)	< 0.001	1.66 (1.46–1.89)	< 0.001	1.92 (1.60–2.29)	< 0.001	1.30 (1.06–1.58)	0.010
Propensity score-matched population												
All-cause death	2.08 (1.74–2.47)	< 0.0001	2.21 (1.83–2.66)	< 0.001	2.18 (1.77–2.69)	< 0.001	2.34 (1.87–2.92)	< 0.001	1.85 (1.34–2.54)	< 0.001	1.96 (1.39–2.77)	< 0.001
MACCE	1.55 (1.36–1.77)	< 0.001	1.58 (1.38–1.82)	< 0.001	1.65 (1.4–1.94)	< 0.001	1.73 (1.46–2.05)	< 0.001	1.35 (1.06–1.71)	0.015	1.31 (1.02–1.69)	0.033

*ACS* acute coronary syndrome, *STEMI* ST-segment elevation myocardial infarction, *NSTE*-*ACS* non-ST-segment elevation acute coronary syndrome, *MACCE* major adverse cardiovascular and cerebrovascular events, *OR* odds ratio

^a^ After forward stepwise selection, the adjusted variables for all-cause death finally included age, sex, SBP, heart rate, heart failure history, cerebrovascular disease history, Killip class at admission, cardiac arrest at admission, eGFR, in-hospital treatment of statins, β-blockers, ACEIs/ARBs, PCI, fivefold elevated myocardial injury markers, type of ACS, and whether patients were transferred from another hospital before the current hospitalization

^b^ After forward stepwise selection, the adjusted variables for MACCE finally included age, sex, smoking, SBP, heart rate, heart failure history, renal failure history, cerebrovascular disease history, Killip class at admission, cardiac arrest at admission, eGFR, in-hospital treatment with dual anti-platelet therapy, statins, ACEIs/ARBs, PCI, fivefold elevated myocardial injury markers, type of ACS, and whether patients were transferred from another hospital before the current hospitalization

We then conducted a sensitivity analysis to evaluate the risk of diabetes/possible diabetes. After excluding patients with possible stress hyperglycemia (n = 2465) in patients without diabetes, diabetes/possible diabetes was still associated with an increased risk of in-hospital all-cause death (OR, 2.22 [95% CI 1.92–2.56]) and MACCE (OR, 1.63 [95% CI 1.46–1.83]).

**Fig. 2:**
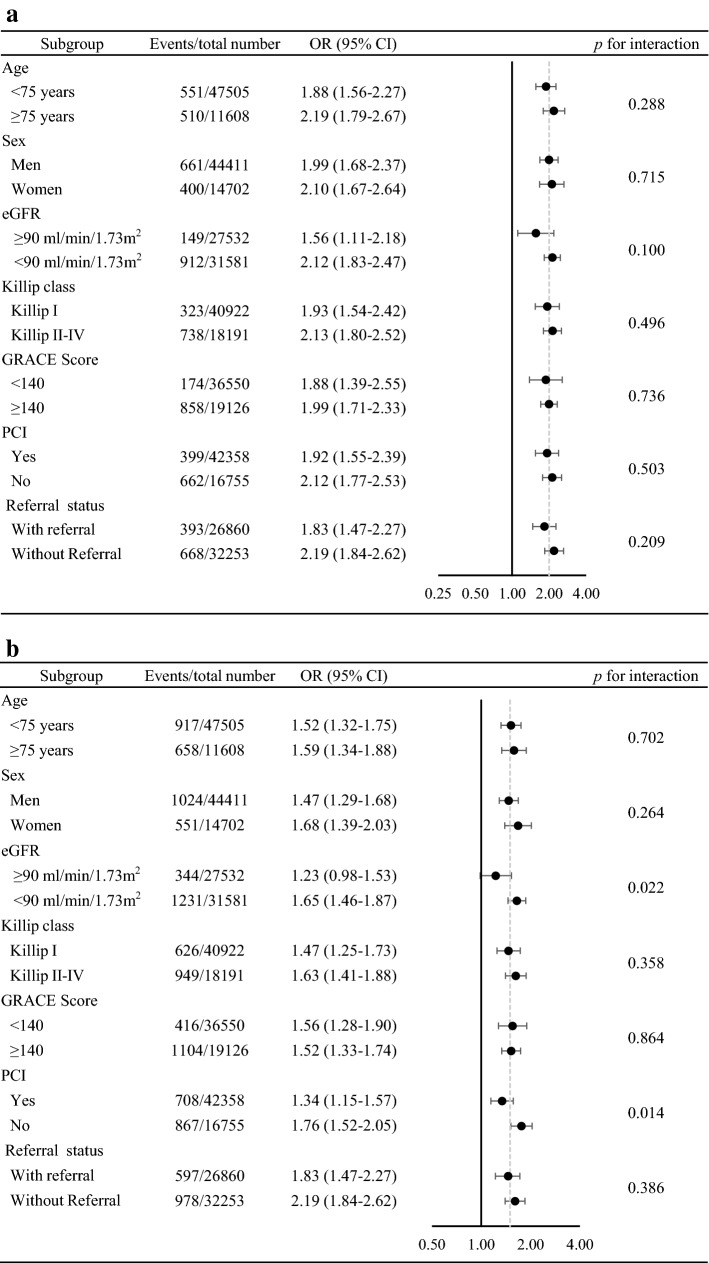
Subgroup analysis for the association between diabetes/possible diabetes and in-hospital outcomes. **a** Association between diabetes/possible diabetes and all-cause death during hospitalization. **b** Association between diabetes/possible diabetes and MACCE during hospitalization. *OR* odds ratio, *eGFR* estimated glomerular filtration rate, *GRACE* Global Registry of Acute Coronary Events, *PCI* percutaneous coronary intervention, *ACS* acute coronary syndrome, *STEMI* ST-segment elevation myocardial infarction, *NSTE*-*ACS* non-ST-segment elevation acute coronary syndrome, *MACCE* major adverse cardiovascular and cerebrovascular events

Subgroup analyses were performed based on important baseline characteristics. Diabetes/possible diabetes was associated with increased risk of all-cause death and MACCE in all subgroups (Fig. 2).

In addition, we conducted a propensity-score-matched analysis to further confirm the association between diabetes/possible diabetes and in-hospital outcomes. After propensity-score matching, 19,315 ACS patients with diabetes/possible diabetes were matched with 19,315 patients without diabetes (patients with possible stress hyperglycemia were excluded before matching). After matching, the standardized differences were less than 10.0% for all variables included for the calculation of propensity score, indicating that ACS patients with and without diabetes/possible diabetes were well matched (Additional file 1: Figure S2). The characteristics and in-hospital treatment between these two groups were re-compared, whereby most of the characteristics were comparable (Additional file 1: Table S4). The rates of all-cause death and MACCE remained higher in patients with diabetes/possible diabetes, and an excess risk of in-hospital outcomes independently associated with diabetes/possible diabetes was also found (all-cause death: OR, 2.21 [95% CI 1.83–2.66]; MACCE: OR, 1.58 [95% CI 1.38–1.82]) (Fig. 1 and Table 4).

## Discussion

In this study, we provided an updated estimation of the burden of diabetes in ACS patients in China and evaluated whether diabetes was independently associated with excess risks for in-hospital all-cause death and MACCE to these patients, based on a nationally representative registry study with a large sample.

### Heavy burden of diabetes among ACS patients

We found that 1 in 3 male ACS patients and 2 in 5 female ACS patients had diabetes/possible diabetes. With the rapid increase in prevalence of diabetes in China, the proportion of diabetes in the ACS patients will continue to rise [15]. The China Heart Study published in 2006 reported that 37.4% of patients with acute coronary artery disease were diagnosed with diabetes by medical history and FBG [7], and 17.4% of these patients were further diagnosed with diabetes by oral glucose tolerance test (OGTT). In this situation, the prevalence of diabetes in ACS patients may be higher than the current prevalence reported herein, as some patients with diabetes may not have been identified because OGTT currently is not applied in the routine clinical workup to assess the diabetic status of patients. These findings indicate that cardiologists in China have to manage a large proportion of ACS patients with diabetes in their clinical care.

However, there remains some doubt about whether our cardiologists are fully prepared to manage this group of patients. In this study, we found that 68.2% of patients (with both measurement of FBG and HbA1c) with FBG ≥ 7.0 mmol/L could be diagnosed with diabetes by HbA1c, which meant that about 70% of patients with possible diabetes could be diagnosed with diabetes with HbA1c tests; however, 57.0% patients did not receive a test for HbA1c during hospitalization. Therefore, for a considerable number of patients the best opportunity to identify and treat their previously undiagnosed diabetes might have been missed, particularly for those patients with little or no routine health care before the occurrence of ACS events. Effectively identifying these patients during hospitalization is thus the first key step in cardiologists’ management strategy. In addition, diabetes also has a great impact on the prognosis of various diseases, and long-term monitoring is necessary [16–18].

### Worse in-hospital outcomes of ACS patients with diabetes

Our study showed that ACS patients with diabetes/possible diabetes had a substantially high risk for in-hospital outcomes compared with patients without diabetes, namely a twofold increased risk of all-cause death and a 1.5-fold increased risk of MACCE. A recently published systematic review and meta-analysis provided a summarized excess risk of early mortality from diabetes status in patients with myocardial infarction/ACS based on 86 studies published from 1970 to 2011 [19]. Here it was reported that diabetes was associated with a 1.7-fold higher risk of early mortality and that the relative risk of early death associated with diabetes did not change over time [19]. Compared with previous studies in Chinese ACS patients, the rates of all-cause death and MACCE during hospitalization have been significantly decreased in our study [20–22]. These findings might suggest that the advancements in the management of ACS patients during the last decades have improved the prognosis of ACS patients but have not led to a reduction of the risk gap between diabetic and non-diabetic patients.

However, one point worth noting is that most of the previous studies did not address the problem of undiagnosed diabetes and stress hyperglycemia [23, 24], which has been defined as possible diabetes in our study. Researchers compared patients with history (previously diagnosed) of diabetes and those without history of diabetes, which included all patients without diabetes, with undiagnosed diabetes, or with stress hyperglycemia. These analyses may underestimate the relative risks of diabetes given the increased risk of the reference group. In our study, we classified patients with undiagnosed diabetes and stress hyperglycemia (FBG ≥ 7 mmol/L) as possible diabetes as they did not have an HbA1c result, and who were associated with a threefold increased risk of all-cause death compared with those without diabetes. Therefore, all ACS patients with FBG ≥ 7 mmol/L or with diabetes should raise major concern in clinical practice in light of their extremely high risk. Relative hyperglycemia, a new concept, reported to associated with complications following an acute myocardial infarction [25], also need to be concerned.

The reasons for the excess risk of all-cause death and MACCE in ACS patients with diabetes/possible diabetes could be partially be explained [26–28], but some reasons are unexplained based on current analysis as the information on anti-diabetic treatment was not available to our study. In our study, we observed that the in-hospital management for ACS was similar between patients with and without diabetes. However, anti-diabetic therapy in the acute phase is also very important for the prognosis of ACS patients with diabetes, and inappropriate hypoglycemic treatment could significantly increase the risk of death [29]. The guidelines have given clear anti-diabetic drug recommendations for patients with both CVD and diabetes [30, 31], and an increasing number of studies have found that newer types of anti-diabetic drugs have a beneficial effect on lowering both blood glucose levels and risks of CVD, but conflicting results still exist [32–36]. In addition, the combined use of anti-diabetic drugs on cardiovascular events should also be concerned [37]. Future studies should take this information into consideration.

Whether in the American, European, or other countries of the world, ACS patients with diabetes is common (usually greater than one-third of patients) and associated with a higher risk of death and other adverse events [2–5, 38]. Although studies have reported that the cardiovascular outcomes of diabetes have been improved in recent years, number of people with diabetes still rises, the absolute burden of CVD will still be high [39]. Effective strategies to better manage the risk of these ACS patients with diabetes and improve their prognosis has always been the focus but also a challenge for cardiologists worldwide. In 2013, the European Society of Cardiology in collaboration with the European Association for the Study of Diabetes developed the second guideline for diabetes, pre-diabetes, and cardiovascular diseases, which calls for physicians in the fields of cardiovascular medicine and diabetes to join forces to research and manage these conditions, given the close relationship between CVD and diabetes [30]. In 2015, the Chinese Society of Cardiology in collaboration with other societies also issued a guideline on the management of abnormal glucose metabolism and CVD [40]. Following the efforts of both cardiologists and diabetologists, the risk of adverse events for ACS patients with diabetes/possible diabetes is expected to decrease [41].

## Limitations

Some limitations of this study are worthy of mention. First, the results of OGTT during hospitalization were unavailable to this study, thus some diabetic patients may have been missed. However, OGTT was not routinely used in clinical practice, which future studies should take into consideration. Second, some patients with only increased FBG could not be definitively diagnosed with diabetes. However, using only tests for FBG revealed that at present, cardiologists do not pay sufficient attention to the diagnosis of diabetes in ACS patients. Finally, as this was a real-world study for ACS patients based on medical records, limited information regarding diabetes was gathered, including incomplete data on body mass index as well as uncollected data on physical exercise information, diabetes types, and in-hospital anti-diabetic therapy. Some other interest points regarding diabetes, such as gender differences, different revascularization strategies, and regional impacts, still need more research in the future [42–44].

## Conclusions

Our results showed that diabetes was highly prevalent among ACS patients in China. Considerable excess risk for early mortality and MACCE was found in ACS patients with diabetes. These findings highlight the importance of early detection and appropriate management of diabetes in ACS patients, using specific therapies that have been demonstrated to improve outcomes.

## Additional file

**Additional file 1: Table S1.** In-hospital outcomes of ACS patients with diabetes/possible diabetes. **Table S2.** The association between diabetes/possible diabetes and in-hospital all-cause death. **Table S3.** The association between diabetes/possible diabetes and in-hospital major adverse cardiovascular and cerebrovascular events. **Table S4.** Characteristics of ACS patients with and without diabetes/ possible diabetes after propensity-score matching. **Table S5.** Investigators of CCC-ACS project. **Figure S1.** Flow chart for study population recruitment. **Figure S2.** Absolute standard differences before and after propensity score matching.

## Abbreviations

CVD: cardiovascular disease
STEMI: ST-segment elevation myocardial infarction
NSTE-ACS: non-ST-segment elevation acute coronary syndrome
ACS: acute coronary syndrome
MACCE: major adverse cardiovascular and cerebrovascular events
HbA1c: glycated hemoglobin A1c
FBG: fasting blood glucose
SBP: systolic blood pressure
DBP: diastolic blood pressure
LDL-C: low-density lipoprotein cholesterol
HDL-C: high-density lipoprotein cholesterol
TG: triglyceride
eGFR: estimated glomerular filtration rate
CHD: coronary heart disease
PCI: percutaneous coronary intervention
CABG: coronary artery bypass grafting
PAD: peripheral artery disease
GRACE: Global Registry of Acute Coronary Events
CI: confidence interval
SD: standard deviation
IQR: interquartile range
ACEIs: angiotensin-converting enzyme inhibitors
ARBs: angiotensin-receptor blockers
OR: odds ratio
OGTT: oral glucose tolerance test
DAPT: dual antiplatelet therapy
UFH: unfractionated heparin
LMWH: low molecular weight heparin
