# Supplementary material for: Prevalence and in-hospital outcomes of diabetes among patients with acute coronary syndrome in China: findings from the Improving Care for Cardiovascular Disease in China-Acute Coronary Syndrome Project
Source: Cardiovasc Diabetol. 2018 Nov 27;17:147. doi: 10.1186/s12933-018-0793-x (PMC6258152; doi:10.1186/s12933-018-0793-x)
Supplement: Supplementary file 1 — Additional file 1: Table S1. In-hospital outcomes of ACS patients with diabetes/possible diabetes. Table S2. The association between diabetes/possible diabetes and in-hospital all-cause death. Table S3. The association between diabetes/possible diabetes and in-hospital major adverse cardiovascular and cerebrovascular events. Table S4. Characteristics of ACS patients with and without diabetes/ possible diabetes after propensity-score matching. Table S5. Investigators of CCC-ACS project. Figure S1. Flow chart for study population recruitment. Figure S2. Absolute standard differences before and after propensity score matching. [file 12933_2018_793_MOESM1_ESM.doc]

**Additional files**

[Table S1 In-hospital outcomes of ACS patients with diabetes/possible diabetes 2](#__RefHeading___Toc527620654)

[Table S2 The association between diabetes/possible diabetes and in-hospital all-cause death 4](#__RefHeading___Toc527620655)

[Table S3 The association between diabetes/possible diabetes and in-hospital major adverse cardiovascular and cerebrovascular events 5](#__RefHeading___Toc527620656)

[Table S4 Characteristics of ACS patients with and without diabetes/ possible diabetes after propensity-score matching 7](#__RefHeading___Toc527620657)

[Table S5 Investigators of CCC-ACS project 9](#__RefHeading___Toc527620658)

[Figure S1 Flow chart for study population recruitment 17](#__RefHeading___Toc527620659)

[Figure S2 Absolute standard differences before and after propensity score matching 18](#__RefHeading___Toc527620660)

# Table S1 In-hospital outcomes of ACS patients with diabetes/possible diabetes

|  | **All ACS** | | **STEMI** | | **NSTE-ACS** | |
| --- | --- | --- | --- | --- | --- | --- |
|  | **Diabetes**  **/possible diabetes** | **No diabetes** | **Diabetes**  **/possible diabetes** | **No diabetes** | **Diabetes**  **/possible diabetes** | **No diabetes** |
| **The whole study population** | | | | | | |
| *N* | 23880 | 39570 | 14650 | 25143 | 9230 | 14427 |
| All-cause death, n (%) | 729 (3.1 [2.8-3.3]) | 494 (1.3 [1.1-1.4]) | 527 (3.6 [3.3-3.9]) | 378 (1.5 [1.4-1.7]) | 202 (2.2 [1.9-2.5]) | 116 (0.8 [0.7-1]) |
| MACCE, n (%) | 944 (4.0 [3.7-4.2]) | 807 (2.0 [1.9-2.2]) | 672 (4.6 [4.3-4.9]) | 582 (2.3 [2.1-2.5]) | 272 (3.0 [2.6-3.3]) | 225 (1.6 [1.4-1.8]) |
| Cardiac death, n (%) | 674 (2.8 [2.6-3]) | 459 (1.2 [1.1-1.3]) | 496 (3.4 [3.1-3.7]) | 358 (1.4 [1.3-1.6]) | 178 (1.9 [1.7-2.2]) | 101 (0.7 [0.6-0.9]) |
| Stent thrombosis, n (%) | 52 (0.2 [0.2-0.3]) | 64 (0.2 [0.1-0.2]) | 43 (0.3 [0.2-0.4]) | 48 (0.2 [0.1-0.3]) | 9 (0.1 [0-0.2]) | 16 (0.1 [0.1-0.2]) |
| Recurrent myocardial infarction, n (%) | 94 (0.4 [0.3-0.5]) | 128 (0.3 [0.3-0.4]) | 64 (0.4 [0.3-0.6]) | 88 (0.4 [0.3-0.4]) | 30 (0.3 [0.2-0.5]) | 40 (0.3 [0.2-0.4]) |
| Stroke, n (%) | 209 (0.9 [0.8-1]) | 224 (0.6 [0.5-0.7]) | 137 (0.9 [0.8-1.1]) | 142 (0.6 [0.5-0.7]) | 72 (0.8 [0.6-1]) | 82 (0.6 [0.5-0.7]) |
| **Propensity score-matched population** | | | | | | |
| *N* | 19315 | 19315 | 11936 | 11910 | 7379 | 7405 |
| All-cause death, n (%) | 386 (2.0 [1.8-2.2]) | 188 (1.0 [0.8-1.1]) | 278 (2.3 [2.1-2.6]) | 129 (1.1 [0.9-1.3]) | 108 (1.5 [1.2-1.8]) | 59 (0.8 [0.6-1.0]) |
| MACCE, n (%) | 557 (2.9 [2.7-3.1]) | 363 (1.9 [1.7-2.1]) | 398 (3.3 [3.0-3.7]) | 244 (2.1 [1.8-2.3]) | 159 (2.2 [1.8-2.5]) | 119 (1.6 [1.3-1.9]) |
| Cardiac death, n (%) | 354 (1.8 [1.7-2.0]) | 172 (0.9 [0.8-1.0]) | 257 (2.2 [1.9-2.4]) | 122 (1 [0.9-1.2]) | 97 (1.3 [1.1-1.6]) | 50 (0.7 [0.5-0.9]) |
| Stent thrombosis, n (%) | 40 (0.2 [0.2-0.3]) | 35 (0.2 [0.1-0.3]) | 33 (0.3 [0.2-0.4]) | 26 (0.2 [0.1-0.3]) | 7 (0.1 [0-0.2]) | 9 (0.1 [0.1-0.2]) |
| Recurrent myocardial infarction, n (%) | 66 (0.3 [0.3-0.4]) | 67 (0.4 [0.3-0.4]) | 43 (0.4 [0.3-0.5]) | 47 (0.4 [0.3-0.5]) | 23 (0.3 [0.2-0.5]) | 20 (0.3 [0.2-0.4]) |
| Stroke, n (%) | 146 (0.8 [0.6-0.9]) | 117 (0.6 [0.5-0.7]) | 102 (0.9 [0.7-1.0]) | 69 (0.6 [0.5-0.7]) | 44 (0.6 [0.4-0.8]) | 48 (0.7 [0.5-0.9]) |

Abbreviation: MACCE: Major adverse cardiovascular and cerebrovascular event; ACS: acute coronary syndrome; STEMI: ST-segment elevation myocardial infarction; NSTE-ACS: non-ST-segment elevation acute coronary syndrome

# Table S2 The association between diabetes/possible diabetes and in-hospital all-cause death

|  | **All ACS** | | **STEMI patients** | | **NSTE-ACS patients** | |
| --- | --- | --- | --- | --- | --- | --- |
|  | **OR (95% CI)** | ***p* value** | **OR (95% CI)** | ***p* value** | **OR (95% CI)** | ***p* value** |
| Diabetes/possible diabetes | 2.04 (1.78-2.33) | <0.001 | 2.07 (1.76-2.43) | <0.001 | 1.93 (1.48-2.51) | <0.001 |
| Age | 1.04 (1.03-1.04) | <0.001 | 1.04 (1.03-1.04) | <0.001 | 1.03 (1.02-1.05) | <0.001 |
| Female | 1.06 (0.92-1.22) | 0.431 | 1.17 (0.99-1.38) | 0.072 | 0.83 (0.64-1.08) | 0.172 |
| Systolic BP | 0.99 (0.99-0.99) | <0.001 | 0.99 (0.99-1.00) | <0.001 | 0.99 (0.98-0.99) | <0.001 |
| Heart rate | 1.01 (1.01-1.02) | <0.001 | 1.02 (1.01-1.02) | <0.001 | 1.01 (1.00-1.02) | 0.003 |
| Heart failure history | 1.36 (1.05-1.77) | 0.020 | 1.40 (0.95-2.07) | 0.093 | 1.29 (0.90-1.85) | 0.173 |
| Cerebrovascular disease history | 1.33 (1.12-1.58) | 0.001 | 1.23 (0.99-1.53) | 0.058 | 1.54 (1.14-2.07) | 0.005 |
| Killip class |  |  |  |  |  |  |
| II-III | 1.51 (1.29-1.78) | <0.001 | 1.51 (1.25-1.83) | <0.001 | 1.56 (1.14-2.13) | 0.006 |
| IV | 3.43 (2.83-4.15) | <0.001 | 3.21 (2.57-4.01) | <0.001 | 4.33 (2.98-6.30) | <0.001 |
| Cardiac arrest at admission | 5.35 (4.34-6.60) | <0.001 | 5.40 (4.28-6.82) | <0.001 | 5.19 (3.11-8.67) | <0.001 |
| eGFR | 0.99 (0.98-0.99) | <0.001 | 0.99 (0.98-0.99) | <0.001 | 0.99 (0.98-0.99) | <0.001 |
| Statins | 0.49 (0.41-0.60) | <0.001 | 0.44 (0.35-0.55) | <0.001 | 0.66 (0.46-0.94) | 0.022 |
| Beta-blockers | 0.83 (0.72-0.97) | 0.015 | 0.77 (0.64-0.92) | 0.004 | 0.99 (0.76-1.29) | 0.930 |
| ACEI/ARB | 0.85 (0.73-0.99) | 0.039 | 0.87 (0.72-1.05) | 0.140 | 0.81 (0.61-1.07) | 0.129 |
| PCI | 0.35 (0.30-0.41) | <0.001 | 0.37 (0.32-0.44) | <0.001 | 0.30 (0.22-0.41) | <0.001 |
| 5-fold elevated myocardial injury markers | 1.95 (1.61-2.37) | <0.001 | 1.50 (1.18-1.89) | 0.001 | 3.05 (2.17-4.28) | <0.001 |
| STEMI | 2.05 (1.75-2.41) | <0.001 | - | - | - | - |
| Patients with referral | 0.70 (0.61-0.81) | <0.001 | 0.70 (0.59-0.82) | <0.001 | 0.69 (0.51-0.93) | 0.013 |

Abbreviation: ACS: acute coronary syndrome; STEMI: ST-segment elevation myocardial infarction; NSTE-ACS: non-ST-segment elevation acute coronary syndrome; OR: odds ratio; BP: blood pressure; eGFR: estimated glomerular filtration rate; ACEI: angiotensin-converting enzyme inhibitors; ARB: angiotensin -receptor blocker; PCI: percutaneous coronary intervention

# Table S3 The association between diabetes/possible diabetes and in-hospital major adverse cardiovascular and cerebrovascular events

|  | **All ACS** | | **STEMI patients** | | **NSTE-ACS patients** | |
| --- | --- | --- | --- | --- | --- | --- |
|  | **OR (95% CI)** | ***p* value** | **OR (95% CI)** | ***p* value** | **OR (95% CI)** | ***p* value** |
| Diabetes/possible diabetes | 1.54 (1.39-1.72) | <0.001 | 1.66 (1.46-1.89) | <0.001 | 1.30 (1.06-1.58) | 0.010 |
| Age | 1.02 (1.02-1.03) | <0.001 | 1.02 (1.02-1.03) | <0.001 | 1.02 (1.01-1.03) | 0.001 |
| Female | 1.05 (0.93-1.18) | 0.460 | 1.13 (0.98-1.31) | 0.099 | 0.89 (0.72-1.11) | 0.303 |
| Smoke | 0.87 (0.77-0.99) | 0.037 | 0.91 (0.78-1.05) | 0.200 | 0.81 (0.63-1.03) | 0.086 |
| Systolic BP | 0.99 (0.99-1.00) | <0.001 | 0.99 (0.99-1.00) | <0.001 | 0.99 (0.99-1.00) | <0.001 |
| Heart rate | 1.01 (1.01-1.01) | <0.001 | 1.01 (1.01-1.02) | <0.001 | 1.01 (1.00-1.01) | 0.008 |
| CHD history | 1.18 (1.01-1.38) | 0.041 | 1.21 (0.98-1.50) | 0.084 | 1.14 (0.90-1.44) | 0.272 |
| Heart failure history | 1.51 (1.21-1.90) | <0.001 | 1.64 (1.16-2.31) | 0.005 | 1.50 (1.10-2.03) | 0.010 |
| Renal failure history | 1.31 (1.02-1.68) | 0.038 | 1.62 (1.15-2.29) | 0.006 | 1.07 (0.74-1.57) | 0.716 |
| Cerebrovascular disease history | 1.40 (1.22-1.62) | <0.001 | 1.37 (1.15-1.64) | 0.001 | 1.47 (1.16-1.87) | 0.001 |
| Killip class |  |  |  |  |  |  |
| II-III | 1.34 (1.18-1.52) | <0.001 | 1.31 (1.13-1.53) | 0.001 | 1.38 (1.10-1.73) | 0.006 |
| IV | 2.84 (2.42-3.33) | <0.001 | 2.71 (2.25-3.28) | <0.001 | 3.28 (2.41-4.46) | <0.001 |
| Cardiac arrest at admission | 4.32 (3.57-5.21) | <0.001 | 4.56 (3.70-5.62) | <0.001 | 3.38 (2.08-5.48) | <0.001 |
| eGFR | 0.99 (0.99-0.99) | <0.001 | 0.99 (0.99-0.99) | <0.001 | 0.99 (0.99-0.99) | <0.001 |
| DAPT | 0.90 (0.83-0.97) | 0.005 | 0.9 (0.81-0.99) | 0.031 | 0.89 (0.79-1.00) | 0.042 |
| Statins | 0.64 (0.54-0.77) | <0.001 | 0.61 (0.49-0.77) | <0.001 | 0.74 (0.54-1.01) | 0.057 |
| ACEI/ARB | 0.83 (0.74-0.93) | 0.002 | 0.83 (0.72-0.96) | 0.0101 | 0.82 (0.67-1.01) | 0.064 |
| PCI | 0.45 (0.40-0.50) | <0.001 | 0.44 (0.39-0.51) | <0.001 | 0.47 (0.38-0.59) | <0.001 |
| 5-fold elevated myocardial injury markers | 1.82 (1.57-2.12) | <0.001 | 1.52 (1.25-1.83) | <0.001 | 2.42 (1.91-3.06) | <0.001 |
| STEMI | 1.79 (1.57-2.03) | <0.001 | - | - | - | - |
| Patients with referral | 0.74 (0.66-0.83) | <0.001 | 0.72 (0.64-0.82) | <0.001 | 0.77 (0.62-0.96) | 0.022 |

Abbreviation: ACS: acute coronary syndrome; STEMI: ST-segment elevation myocardial infarction; NSTE-ACS: non-ST-segment elevation acute coronary syndrome; OR: odds ratio; BP: blood pressure; eGFR: estimated glomerular filtration rate; DAPT: dual antiplatelet therapy; ACEI: angiotensin-converting enzyme inhibitors; ARB: angiotensin -receptor blocker; PCI: percutaneous coronary intervention

# Table S4 Characteristics of ACS patients with and without diabetes/ possible diabetes after propensity-score matching

|  | **Diabetes/**  **possible diabetes**  **(N=19315)** | **No diabetes**  **(N=19315)** | ***p* value** |
| --- | --- | --- | --- |
| Age, mean (SD), years | 63.7 (11.8) | 63.9 (12.7) | 0.136 |
| Female, n (%) | 5311 (27.5) | 5361 (27.8) | 0.569 |
| Current smoking, n (%) | 7827 (40.5) | 7777 (40.3) | 0.604 |
| Heart rate, mean (SD), bpm | 78.2 (15.9) | 78.4 (16.1) | 0.139 |
| Systolic BP, mean (SD), mmHg | 131.3 (23.7) | 131.4 (23.5) | 0.698 |
| Diastolic BP, mean (SD), mmHg | 77.7 (14.2) | 78.8 (14.3) | <0.001 |
| LDL-C, mean (SD), mg/dl | 105.9 (38.6) | 106.1 (38.2) | 0.633 |
| HDL-C, mean (SD), mg/dl | 41.2 (16.2) | 41.5 (14.5) | 0.013 |
| TG, median (IQR), mg/dl | 131.9 (93.9-189.5) | 125.7 (90.3-185.1) | <0.001 |
| eGFR, median (IQR), ml/min/1.73m2 | 87.9 (67.3-99.9) | 86.7 (68.5-98.4) | <0.001 |
| Medical history |  |  |  |
| Myocardial infarction, n (%) | 1615 (8.4) | 1656 (8.6) | 0.454 |
| PCI, n (%) | 1634 (8.5) | 1666 (8.6) | 0.560 |
| CABG, n (%) | 114 (0.6) | 110 (0.6) | 0.789 |
| Cerebrovascular disease, n (%) | 2075 (10.7) | 2140 (11.1) | 0.289 |
| PAD, n (%) | 191 (1.0) | 210 (1.1) | 0.340 |
| Heart failure, n (%) | 400 (2.1) | 415 (2.2) | 0.595 |
| Renal failure, n (%) | 308 (1.6) | 330 (1.7) | 0.380 |
| Killip class, n (%) |  |  | 0.186 |
| I | 13144 (68.1) | 13017 (67.4) |  |
| II-IV | 5181 (26.8) | 5337 (27.6) |  |
| IV | 990 (5.1) | 961 (5.0) |  |
| Condition at admission |  |  |  |
| Heart failure ^a^, n (%) | 1923 (10.0) | 1724 (8.9) | 0.001 |
| Cardiac shock ^b^, n (%) | 565 (2.9) | 531 (2.8) | 0.299 |
| Cardiac arrest, n (%) | 328 (1.7) | 336 (1.7) | 0.754 |
| 5-fold elevated myocardial injury markers^c^, n (%) | 13673 (72.7) | 13019 (69.4) | <0.001 |
| GRACE score≥140 ^d^, n (%) | 6601 (36.3) | 6532 (36.1) | 0.697 |
| Type of ACS, n (%) |  |  | 0.786 |
| STEMI | 11910(61.7) | 11936(61.8) |  |
| NSTE-ACS | 7405(38.3) | 7379(38.2) |  |
| In-hospital treatment |  |  |  |
| DAPT, n (%) | 17973 (93.7) | 17877 (93.4) | 0.158 |
| Aspirin, n (%) | 18320 (95.5) | 18280 (95.5) | 0.780 |
| P2Y_12_ inhibitors, n (%) | 18498 (95.9) | 18427 (95.5) | 0.058 |
| GPIIb/IIIa, n (%) | 6241 (32.4) | 5792 (30) | <0.001 |
| Anticoagulant, n (%) | 14664 (76) | 14577 (75.5) | 0.281 |
| UFH | 795 (4.1) | 672 (3.5) | 0.001 |
| LMWH | 13446 (69.8) | 13536 (70.3) | 0.340 |
| Fondaparinux sodium | 321 (1.7) | 278 (1.4) | 0.076 |
| Other anticoagulant | 393 (2.0) | 365 (1.9) | 0.302 |
| Statins, n (%) | 18168 (94.2) | 18216 (94.5) | 0.290 |
| Beta-blockers, n (%) | 4250 (66.5) | 4081 (67.2) | 0.626 |
| ACEI/ARB, n (%) | 9471 (51.6) | 9270 (50.4) | 0.019 |
| PCI, n (%) | 13924 (72.1) | 13460 (69.7) | <0.001 |
| Time of PCI ^e^, n (%) |  |  | <0.001 |
| <2 h | 4632 (41.0) | 4175 (38.4) |  |
| 2-11.9 h | 1253 (11.1) | 1050 (9.7) |  |
| 12-23.9 h | 526 (4.7) | 474 (4.4) |  |
| 24-71.9 h | 1824 (16.1) | 1904 (17.5) |  |
| ≥72 h | 3067 (27.1) | 3273 (30.1) |  |
| Stents, n (%) | 12414 (64.3) | 11758 (60.9) | <0.001 |
| Type of stents ^f^, n (%) |  |  | 0.420 |
| Drug eluting stent | 11874 (97.7) | 11269 (97.9) |  |
| Bare metal stent | 159 (1.3) | 150 (1.3) |  |
| Other | 120 (1.0) | 95 (0.8) |  |
| CABG, n (%) | 143 (0.7) | 136 (0.7) | 0.674 |

Abbreviation: ACS: acute coronary syndrome; BP: blood pressure; LDL-C: low-density lipoprotein cholesterol; HDL-C: high-density lipoprotein cholesterol; TG: triglyceride; eGFR: estimated glomerular filtration rate; CHD: coronary heart disease; PAD: peripheral artery disease; GRACE: Global Registry of Acute Coronary Events; STEMI: ST-segment elevation myocardial infarction; NSTE-ACS: non-ST-segment elevation acute coronary syndrome; DAPT: dual antiplatelet therapy; UFH: unfractionated heparin; LMWH: low molecular weight heparin; ACEI: angiotensin-converting enzyme inhibitors; ARB: angiotensin -receptor blocker; PCI: percutaneous coronary intervention; CABG: coronary artery bypass grafting

^a^ Heart failure, data of cardiac failure were not available for 24 patients

^b^ Cardiac shock, data of cardiac shock were not available for 14 patients

^c^ 5-fold elevated myocardial injury markers, data of myocardial injury markers were not available for1063 patients

^d^ GRACE score, data of cardiac arrest were not available for 2334 patients

^e^ Time of PCI, time from admission to PCI, detailed data of time of PCI was not available for 5206 patients with PCI

^f^ Type of stents, data of type of stents were not available for 505 patients with stent implantation

# Table S5 Investigators of CCC-ACS project

| **ID** | **Hospitals** | **Territories** | **Provinces** | **City** | **Investigator** |
| --- | --- | --- | --- | --- | --- |
| 1 | Shanxi Cardiovascular Hospital | Northern China | Shanxi | Taiyuan | Bao Li |
| 2 | Nanjing Drum Tower Hospital, The Affiliated Hospital of Nanjing University Medical School | Eastern China | Jiangsu | Nanjing | Biao Xu, Guangshu Han |
| 3 | Hainan General Hospital | Southern China | Hainan | Haikou | Bin Li |
| 4 | The Second Hospital of Jilin University | Northeast China | Jilin | Changchun | Bin Liu |
| 5 | The 2nd Affiliated Hospital of Harbin Medical University | Northeast China | Heilongjiang | Harbin | Bo Yu |
| 6 | The Ninth Hospital Affiliated to Shanghai Jiaotong University School of Medicine | Eastern China | Shanghai | Shanghai | Changqian Wang |
| 7 | Henan Provincial People's Hospital | Central China | Henan | Zhengzhou | Chuanyu Gao |
| 8 | Shanxi Provincial People's Hospital | Northern China | Shanxi | Taiyuan | Chunlin Lai |
| 9 | Xinqiao Hospital, Third Military Medical University | Southwest China | Chongqing | Chongqing | Cui Bin, Lan Huang |
| 10 | China Meitan General Hospital | Northern China | Beijing | Beijing | Di Wu |
| 11 | The 309th Hospital of Chinese People's Liberation Army | Northern China | Beijing | Beijing | Fakuan Tang, Jun Xiao |
| 12 | Zhongda Hospital, Southeast University | Eastern China | Jiangsu | Nanjing | Genshan Ma |
| 13 | The First Affiliated Hospital of Liaoning Medical University | Northeast China | Liaoning | Jinzhou | Guizhou Tao |
| 14 | Xinjiang Uygur Autonomous Region People’s Hospital | Northwest China | Xinjiang | Urumchi | Guoqing Li |
| 15 | Sir Run Run Shaw Hospital, College of Medicine, Zhejiang University | Eastern China | Zhejiang | Hangzhou | Guosheng Fu |
| 16 | Beijing Friendship Hospital, Capital Medical University | Northern China | Beijing | Beijing | Hongwei Li |
| 17 | The First Affiliated Hospital of Bengbu Medical College | Eastern China | Anhui | Bengbu | Honhju Wang |
| 18 | General Hospital of TISCO | Northern China | Shanxi | Taiyuan | Huifeng Wang |
| 19 | Dongguan People's Hospital | Southern China | Guangdong | Dongguan | Jianfeng Ye |
| 20 | Panyu Hospital of Chinese Medicine | Southern China | Guangdong | Guangzhou | Jianhao Li |
| 21 | Peking University First Hospital | Northern China | Beijing | Beijing | Jie Jiang |
| 22 | Sun Yat-sen Memorial Hospital, Sun Yat-sen University | Southern China | Guangdong | Guangzhou | Jingfeng Wang |
| 23 | Guangdong General Hospital | Southern China | Guangdong | Guangzhou | Jiyan Chen |
| 24 | Hospital of Xinjiang Production & Construction Corps | Northwest China | Xinjiang | Urumchi | Junming Liu |
| 25 | The Military General Hospital of Beijing PLA | Northern China | Beijing | Beijing | Junxia Li |
| 26 | The First Affiliated Hospital of Guangxi Medical University | Southern China | Guangxi | Nanning | Lang Li |
| 27 | Tongren Hospital Affiliated to Shanghai Jiaotong University School of Medicine | Eastern China | Shanghai | Shanghai | Li Jiang |
| 28 | Binzou City Center Hospital | Eastern China | Shandong | Binzhou | Lijun Meng |
| 29 | The First Affiliated Hospital of Zhengzhou University | Central China | Henan | Zhengzhou | Ling Li |
| 30 | Xijing Hospital | Northwest China | Shaanxi | Xi'an | Ling Tao |
| 31 | The Affiliated Hospital of Guizhou Medical University | Southwest China | Guizhou | Guiyang | Lirong Wu |
| 32 | First Affiliated Hospital of the People's Liberation Army General Hospital | Northern China | Beijing | Beijing | Miao Tian |
| 33 | The Second People's Hospital of Yunnan Province | Southwest China | Yunnan | Kunming | Minghua Han |
| 34 | Haikou People's Hospital | Southern China | Hainan | Haikou | Moshui Chen |
| 35 | Gansu Provincial Hospital | Northwest China | Gansu | Lanzhou | Ping Xie |
| 36 | The First Affiliated Hospital of Henan University of Science and Technology | Central China | Henan | Luoyang | Pingshuan Dong |
| 37 | Chenzhou First People's Hospital | Central China | Hunan | Chenzhou | Qiaoqing Zhong |
| 38 | People’s Hospital of Qinghai Province | Northwest China | Qinghai | Xining | Rong Chang |
| 39 | Affiliated Hospital of Ningxia Medical University | Northwest China | Ningxia | Yinchuan | Shaobin Jia |
| 40 | Beijing Anzhen Hospital, Capital Medical University | Northern China | Beijing | Beijing | Shaoping Nie, Xiaohui Liu |
| 41 | North Jiangsu People's Hospital | Eastern China | Jiangsu | Yangzhou | Shenghu He |
| 42 | Shanghai Sixth People's Hospital | Eastern China | Shanghai | Shanghai | Shixin Ma |
| 43 | The First Hospital of Handan | Northern China | Hebei | Handan | Shuanli Xin |
| 44 | Huai'an First People's Hospital | Eastern China | Jiangsu | Huai'an | Shuren Ma |
| 45 | The First Affiliated Hospital of Chongqing Medical University | Southwest China | Chongqing | Chongqing | Suxin Luo |
| 46 | Navy General Hospital | Northern China | Beijing | Beijing | Tianchang Li |
| 47 | Zhejiang Provincial Hospital of TCM | Eastern China | Zhejiang | Hangzhou | Wei Mao |
| 48 | The Third Xiangya Hospital of Central South University | Central China | Hunan | Changsha | Weihong Jiang |
| 49 | Affiliated Hospital of Qinghai University | Northwest China | Qinghai | Xining | Weijun Liu |
| 50 | Teda International Cardiovascular Hospital | Northern China | Tianjin | Tianjin | Wenhua Lin |
| 51 | The Second Hospital of Hebei Medical University | Northern China | Hebei | Shijiazhuang | Xianghua Fu |
| 52 | Changhai Hospital of Shanghai | Eastern China | Shanghai | Shanghai | Xianxian Zhao |
| 53 | The Second Affiliated Hospital to Nanchang University | Eastern China | Jiangxi | Nanchang | Xiaoshu Cheng |
| 54 | Hebei General Hospital | Northern China | Hebei | Shijiazhuang | Xiaoyong Qi |
| 55 | Inner Mongolia People's Hospital | Northern China | Inner Mongolia | Hohhot | Xingsheng Zhao |
| 56 | The General Hospital of Shenyang Military Region | Northeast China | Liaoning | Shenyang | Yaling Han |
| 57 | The First Hospital of Jilin University | Northeast China | Jilin | Changchun | Yang Zheng |
| 58 | Tianjin Chest Hospital | Northern China | Tianjin | Tianjin | Yin Liu |
| 59 | Hunan Provincial People's Hospital | Central China | Hunan | Changsha | Ying Guo |
| 60 | People's Hospital of Yuxi City | Southwest China | Yunnan | Yuxi | Yinglu Hao |
| 61 | The People's Hospital of Guangxi Zhuang Autonomous Region | Southern China | Guangxi | Nanning | Yingzhong Lin |
| 62 | The First Teaching Hospital of Xinjiang Medical University | Northwest China | Xinjiang | Urumchi | Yitong Ma |
| 63 | Baogang Hospital | Northern China | Inner Mongolia | Baotou | Yongdong Li |
| 64 | Tianjin Medical University General Hospital | Northern China | Tianjin | Tianjin | Yuemin Sun |
| 65 | The Second Affiliated Hospital of Zhengzhou University | Central China | Henan | Zhengzhou | Yulan Zhao |
| 66 | Nanfang Hospital of Southern Medical University | Southern China | Guangdong | Guangzhou | Yuqing Hou |
| 67 | The First Affiliated Hospital to Nanchang University | Eastern China | Jiangxi | Nanchang | Zeqi Zheng |
| 68 | The First Affiliated Hospital of Lanzhou University | Northwest China | Gansu | Lanzhou | Zheng Zhang |
| 69 | The Third Hospital of Shijiazhuang | Northern China | Hebei | Shijiazhuang | Zhenguo Ji |
| 70 | Wuxi People's Hospital | Eastern China | Jiangsu | Wuxi | Zhenyu Yang |
| 71 | Jiangsu Province Hospital | Eastern China | Jiangsu | Nanjing | Zhijian Yang |
| 72 | The Second Hospital of Shanxi Medical University | Northern China | Shanxi | Taiyuan | Zhiming Yang |
| 73 | The Affiliated Hospital of Xuzhou Medical College | Eastern China | Jiangsu | Xuzhou | Zhirong Wang |
| 74 | Southwest Hospital, Third Military Medical University | Southwest China | Chongqing | Chongqing | Zhiyuan Song |
| 75 | The First Affiliated Hospital of Xi’an Jiaotong University | Northwest China | Shaanxi | Xi'an | Zuyi Yuan |
| 76 | Yangzhou First People's Hospital | Eastern China | Jiangsu | Yangzhou | Aihua Li |
| 77 | Hospital 463 of Chinese People's Liberation Army | Northeast China | Liaoning | Shenyang | Bosong Yang |
| 78 | The Central Hospital of Mianyang | Northwest China | Sichuan | Mianyang | Caidong Luo |
| 79 | Liaocheng People's Hospital | Eastern China | Shandong | Liaocheng | Chunyan Zhang |
| 80 | Yancheng Third People's Hospital | Eastern China | Jiangsu | Yancheng | Chunyang Wu |
| 81 | The Second Xiangya Hospital of Central South University | Central China | Hunan | Changsha | Daoquan Peng |
| 82 | The Central Hospital of Panzhihua | Northwest China | Sichuan | Panzhihua | Dawen Xu |
| 83 | The First Hospital of Qiqihaer City | Northeast China | Heilongjiang | Qiqihaer | Gang Xu |
| 84 | The Third the People‘s Hospital of Bengbu | Eastern China | Anhui | Bengbu | Gengsheng Sang |
| 85 | The First Hospital of Jiamusi | Northeast China | Heilongjiang | Jiamusi | Guixia Zhang |
| 86 | Zhoushan People's Hospital | Eastern China | Zhejiang | Zhoushan | Guoxiong Chen |
| 87 | Dalian Municipal Central Hospital | Northeast China | Liaoning | Dalian | Hailong Lin |
| 88 | Renmin Hospital of Wuhan University | Central China | Hubei | Wuhan | Hong Jiang |
| 89 | Ningxia People's Hospital | Northwest China | Ningxia | Yinchuan | Hong Luan |
| 90 | The First People's Hospital of Yunnan Province (Kunhua Hospital) | Northwest China | Yunnan | Kunming | Hong Zhang |
| 91 | The Central Hospital of Zhoukou | Central China | Henan | Zhoukou | Hualing Liu |
| 92 | Anyang District Hospital | Central China | Henan | Anyang | Hui Liu |
| 93 | Sichuan Provincial People’s Hospital | Northwest China | Sichuan | Chengdu | Jianhong Tao |
| 94 | Mudanjiang Cardiovascular Disease Hospital | Northeast China | Heilongjiang | Mudanjiang | Jianwen Liu |
| 95 | Yichang Central Hospital | Central China | Hubei | Yichang | Jiawang Ding |
| 96 | Qilu Hospital of Shandong University | Eastern China | Shandong | Jinan | Jifu Li |
| 97 | Affiliated Hospital of Jiangsu University | Eastern China | Jiangsu | Zhenjiang | Jinchuan Yan |
| 98 | The First People's Hospital of Nanning City | Southern China | Guangxi | Nanning | Jinru Wei |
| 99 | The First Affiliated Hospital of Fujian Medical University | Eastern China | Fujian | Fuzhou | Jinzi Su |
| 100 | Chengdu Third People’s Hospital | Northwest China | Sichuan | Chengdu | Jiong Tang |
| 101 | Yantaishan hospital | Eastern China | Shandong | Yantai | Juexin Fan |
| 102 | Qingdao Municipal Hospital | Eastern China | Shandong | Qingdao | Jun Guan |
| 103 | Zhongshan Hospital Affiliated to Fudan University | Eastern China | Shanghai | Shanghai | Junbo Ge |
| 104 | Longyan First Hospital | Eastern China | Fujian | Longyan | Kaihong Chen |
| 105 | Affiliated Hospital of Guangdong Medical College | Southern China | Guangdong | Guangzhou | Keng Wu |
| 106 | Jiangxi Provincial People's Hospital | Eastern China | Jiangxi | Nanchang | Lang Ji |
| 107 | Anhui Provincial Hospital | Eastern China | Anhui | Hefei | Likun Ma |
| 108 | Xiangtan City Central Hospital | Central China | Hunan | Xiangtan | Lilong Tang |
| 109 | The First Hospital of Haerbin City | Northeast China | Heilongjiang | Harbin | Lin Wei |
| 110 | Central Hospital Affiliated to Shenyang Medical College | Northeast China | Liaoning | Shenyang | Man Zhang, Kaiming Chen |
| 111 | The Central Hospital of Wuhan | Central China | Hubei | Wuhan | Manhua Chen |
| 112 | Hangzhou First People's Hospital | Eastern China | Zhejiang | Hangzhou | Ningfu Wang |
| 113 | The Central Hospital of Xuzhou | Eastern China | Jiangsu | Xuzhou | Peiying Zhang |
| 114 | The Second hospital of Dalian Medical University | Northeast China | Liaoning | Dalian | Peng Qu |
| 115 | The First Affiliated Hospital of Liaoning University of Traditional Chinese Medicine | Northeast China | Liaoning | Shenyang | Ping Hou |
| 116 | Beijing Tsinghua Changgung Hospital | Northern China | Beijing | Beijing | Ping Zhang |
| 117 | Guizhou Provincial People's Hospital | Northwest China | Guizhou | Guiyang | Qiang Wu |
| 118 | The First Affiliated Hospital of Xiamen University | Eastern China | Fujian | Xiamen | Qiang Xie |
| 119 | Quanzhou First Hospital | Eastern China | Fujian | Quanzhou | Rong Lin |
| 120 | Wuzhou People's Hospital | Southern China | Guangxi | Wuzhou | Shaowu Ye |
| 121 | The Central Hospital of Jilin | Northeast China | Jilin | Changchun | Shuangbin Li |
| 122 | Xiangya Hospital Central South University | Central China | Hunan | Changsha | Tianlun Yang |
| 123 | Guangzhou Red Cross Hospital | Southern China | Guangdong | Guangzhou | Tongguo Wu |
| 124 | The First Affiliated Hospital of Guangzhou Medical College | Southern China | Guangdong | Guangzhou | Wei Wang |
| 125 | The First Affiliated Hospital of Wenzhou Medical University | Eastern China | Zhejiang | Wenzhou | Weijian Huang |
| 126 | The Second Affiliated Hospital of Soochow University | Eastern China | Jiangsu | Suzhou | Weiting Xu |
| 127 | Wuhan Asia Heart Hospital | Central China | Hubei | Wuhan | Xi Su |
| 128 | The First Affiliated Hospital of Soochow University | Eastern China | Jiangsu | Suzhou | Xiangjun Yang |
| 129 | Affiliated Hospital of Yan'an University | Northwest China | Shaanxi | Yan'an | Xiaochuan Ma |
| 130 | The First People's Hospital of Jining | Eastern China | Shandong | Jining | Xiaofei Sun |
| 131 | The Central Hospital of Taiyuan | Northern China | Shanxi | Taiyuan | Xiaoping Chen |
| 132 | West China Hospital of Sichuan University | Northwest China | Sichuan | Chengdu | Xiaoping Chen |
| 133 | The Third Affiliated Hospital of Guangzhou Medical College | Southern China | Guangdong | Guangzhou | Ximing Chen |
| 134 | The First Affiliated Hospital of Wannan Medical College | Eastern China | Anhui | Wuhu | Xingsheng Tang |
| 135 | Tangdu Hospital of The Fourth Military Medical University | Northwest China | Shaanxi | Xi'an | Xue Li |
| 136 | Shanghai East Hospital Affiliated to Tongji University | Eastern China | Shanghai | Shanghai | Xuebo Liu |
| 137 | Xiamen Cardiovascular Disease Hospital | Eastern China | Fujian | Xiamen | Yan Wang |
| 138 | Zhongnan hospital of Wuhan University | Central China | Hubei | Wuhan | Yanggan Wang |
| 139 | Fujian Provincial Hospital | Eastern China | Fujian | Fuzhou | Yansong Guo |
| 140 | The First Affiliated hospital of Dalian Medical University | Northeast China | Liaoning | Dalian | Yanzong Yang |
| 141 | The First People's Hospital of Changde | Central China | Hunan | Changde | Yi Huang |
| 142 | The First Affiliated Hospital of China Medical University | Northeast China | Liaoning | Shenyang | Yingxian Sun |
| 143 | The Fourth Affiliated Hospital of China Medical University | Northeast China | Liaoning | Shenyang | Yuanzhe Jin |
| 144 | Cangzhou Central Hospital | Northern China | Hebei | Cangzhou | Zesheng Xu |
| 145 | The Central Hospital of Shaoyang | Central China | Hunan | Shaoyang | Zewei Ouyang |
| 146 | The People's Hospital of Liaoning Province | Northeast China | Liaoning | Shenyang | Zhanquan Li |
| 147 | The First Affiliated Hospital of Jiamusi University | Northeast China | Heilongjiang | Jiamusi | Zhaofa He |
| 148 | Tangshan Gongren Hospital | Northern China | Hebei | Tangshan | Zheng Ji |
| 149 | Huaibei Miners General Hospital | Eastern China | Anhui | Huaibei | Zhenqi Su |
| 150 | Linyi People's Hospital | Eastern China | Shandong | Linyi | Zhihong Ou |


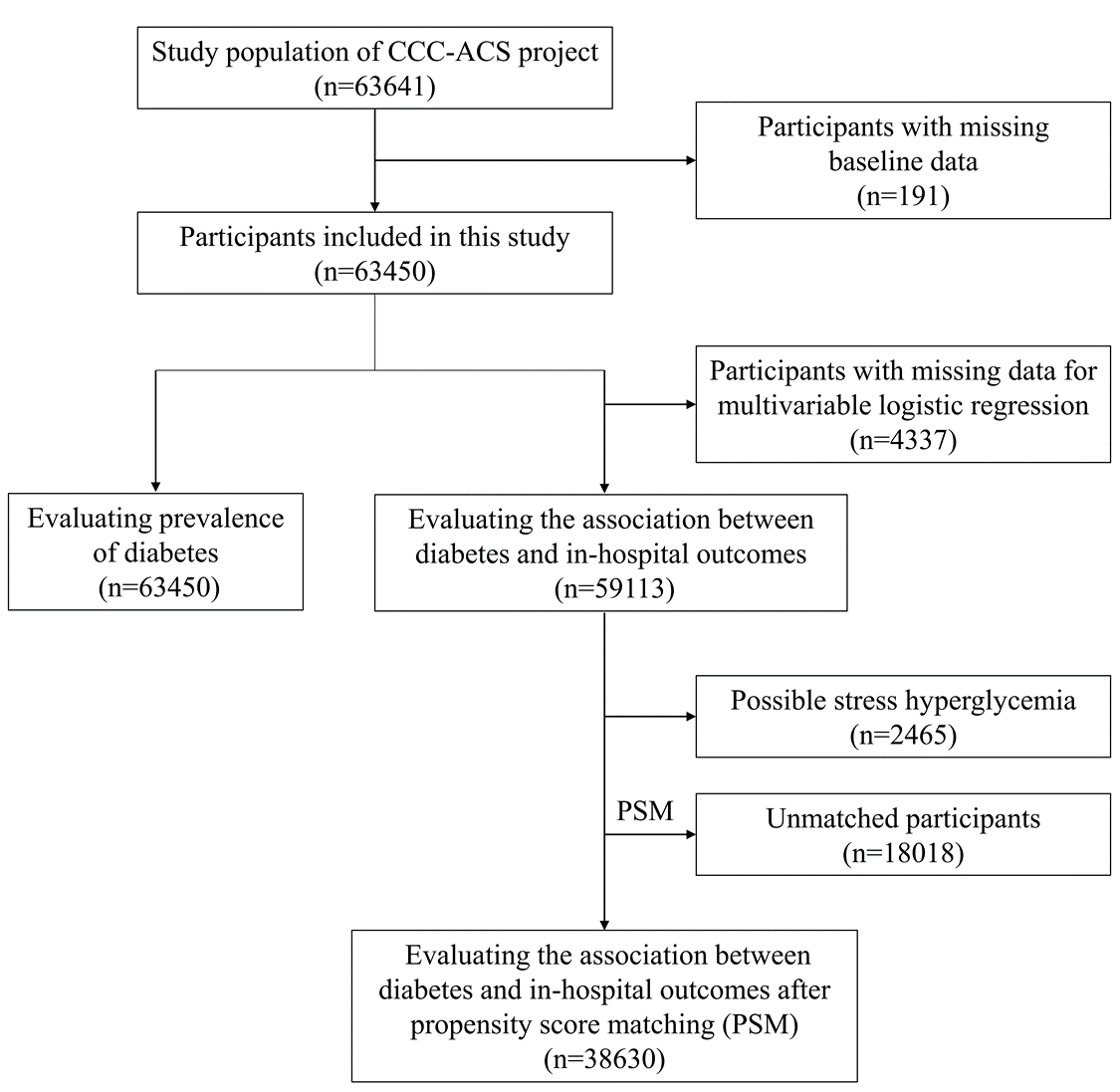


# Figure S1 Flow chart for study population recruitment


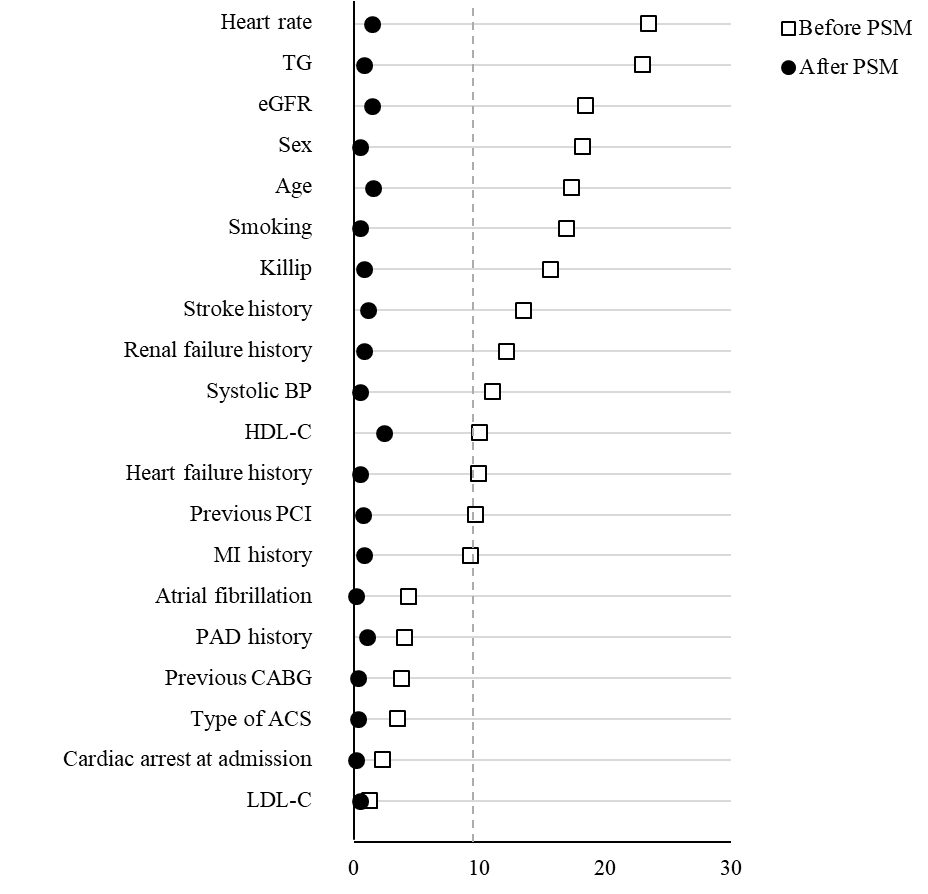


# Figure S2 Absolute standard differences before and after propensity score matching
